# Associations between use of aspirin and magnetic resonance imaging‐derived liver fat and fibroinflammation

**DOI:** 10.1111/dom.70570

**Published:** 2026-02-18

**Authors:** Qi Feng, Pinelopi Manousou, Chioma N. Izzi‐Engbeaya, Jun Liu, Mark Woodward

**Affiliations:** ^1^ The George Institute for Global Health (UK), School of Public Health, Faculty of Medicine Imperial College London London UK; ^2^ Division of Digestive Diseases, Department of Metabolism, Digestion and Reproduction Faculty of Medicine, Imperial College London London UK; ^3^ Department of Hepatology St Mary's Hospital, Imperial College Healthcare NHS Trust London UK; ^4^ Section of Investigative Medicine and Endocrinology, Department of Metabolism, Digestion and Reproduction, Faculty of Medicine Imperial College London London UK; ^5^ Department of Endocrinology St Mary's Hospital, Imperial College Healthcare NHS Trust London UK; ^6^ Department of Ophthalmology Tongji Hospital, Tongji Medical College, Huazhong University of Science and Technology Wuhan China; ^7^ The George Institute for Gdlobal Health (Australia) University of South New Wales Sydney Australia

**Keywords:** aspirin, cT1, liver fat, liver steatosis, MRI, PDFF

## Abstract

**Background:**

The effects of aspirin on hepatic steatosis and fibroinflammation are unclear. The study aimed to examine the association between aspirin use and liver magnetic resonance imaging (MRI)‐derived liver fat and corrected T1 (cT1).

**Methods:**

We used UK Biobank imaging cohort data. Aspirin use was self‐reported at baseline and imaging assessment, and the main exposures were aspirin use at imaging assessment and longitudinal aspirin use patterns (never users, initiators, discontinuers, vs. persistent users). Outcomes were MRI‐derived liver fat (%) and cT1 (ms). Multivariable adjustment analyses and inverse probability of treatment weighting (IPTW) analyses were performed, accounting for demographic, lifestyle and clinical factors.

**Results:**

We included 36 413 participants (mean age 64.6 years, 51.4% female). Aspirin use at imaging assessment was associated with lower liver fat (−0.35; 95% confidence interval [CI]: −0.51, −0.20) and slightly higher cT1 (5.13; 95% CI: 3.23, 7.03). Analyses on longitudinal aspirin use patterns showed that compared to never users, initiators and persistent users showed lower liver fat (−0.48; −0.69, −0.28) and (−0.24; −0.45, −0.02) and higher cT1 (2.94; 0.38, 5.49) and (8.31; 5.65, 10.97). IPTW analyses showed consistent results.

**Conclusion:**

In this large population‐based cohort, aspirin use was linked to reduced liver fat, but a small, clinically insignificant (i.e., <80 ms) increase in cT1. These findings suggest aspirin may mitigate steatosis through metabolic pathways but does not necessarily rapidly reverse fibroinflammatory injury.

## 1 INTRODUCTION

Aspirin is among the most widely used medications worldwide, primarily for primary and secondary prevention in people with cardiovascular disease (CVD).^1^, ^2^, ^3^ Steatotic liver disease (SLD) affects over one‐third of the global population, making it the most common chronic liver disease.^4^ It is defined by the excessive accumulation of fat in the liver.^5^, ^6^ Beyond its hepatic manifestation, liver steatosis is strongly linked to cardiometabolic risk factors and is associated with various extrahepatic diseases, including CVD, cancers and chronic kidney disease.^7^, ^8^, ^9^

The potential effects of aspirin on liver steatosis have been the focus of increasing research, but the findings have been inconsistent. Early cross‐sectional studies suggested associations between aspirin use and lower prevalence of SLD,^10^ as well as less severe histologic features and lower risk of progression to advanced fibrosis in SLD populations.^11^, ^12^ A recent review further concluded that most studies supported a protective effect of aspirin on SLD prevalence and progression.^13^ In contrast, Huang et al.^14^ reported a positive association between aspirin use and MASLD risk, while a US study found no association between aspirin and liver fibrosis measures in people with myocardial infarction.^15^ Evidence from randomised controlled trials is limited, with one small trial reporting that low‐dose aspirin reduced liver fat content in 80 non‐cirrhotic MASLD patients.^16^

These discrepancies may reflect key limitations of prior research. Firstly, most existing studies relied on clinical diagnoses of SLD from electronic health records, which are prone to underestimation of SLD prevalence and incidence.^17^ Few studies have examined the associations with accurate imaging‐based measures of liver fat percentage and liver disease activity in general populations. Secondly, many previous studies assessed aspirin exposure only cross‐sectionally, without accounting for longitudinal patterns of aspirin use; while, thirdly, their sample sizes have often been modest.

To address these gaps, the present study aimed to investigate the associations of aspirin use and longitudinal use patterns, with imaging‐derived liver fat and liver disease activity in a large set of UK Biobank (UKB) participants.

## 2 MATERIALS AND METHODS

The UKB cohort study comprises approximately half a million individuals recruited between 2006 and 2010. At baseline assessment, participants completed a questionnaire on socio‐demographic background, lifestyle, and medical history. Biological samples of blood, urine and saliva were also collected.^18^ Since 2014, participants have been invited to attend an imaging visit, which included magnetic resonance imaging (MRI) scans of the brain, heart and abdominal organs. As of September 2025, liver MRI data are available for around 40 000 participants.^19^ Baseline data were updated during imaging visits; however, biochemistry data were not available at imaging visits.

We excluded participants who did not have liver MRI data, had withdrawn consent, had chronic liver conditions other than SLD at baseline or at imaging assessment (e.g., viral hepatitis, liver fibrosis, liver cirrhosis, hepatocellular carcinoma, hemochromatosis, Wilson's disease, biliary cirrhosis, autoimmune hepatitis, primary sclerosing cholangitis, toxic liver disease and Budd‐Chiari syndrome), or had missing data in key variables (socio‐demographic, lifestyle, cardiometabolic risk factors).

### 2.1 Exposure and outcome

At both UKB baseline and imaging visits, participants reported whether they regularly used aspirin (yes/no), defined as taking it on most days during the past 4 weeks. The primary exposure was aspirin use at the imaging visit. We assessed the longitudinal pattern of aspirin use based on the responses at baseline and imaging visits, classified as never users, initiators, discontinuers and persistent users. Specifically, “never users” were defined as participants reporting no aspirin use at both baseline and imaging assessments; “initiators” as those reporting no aspirin use at baseline, but aspirin use at the imaging assessment; “discontinuers” as those reporting aspirin use at baseline but not at the imaging assessment; and “persistent users” as those reporting aspirin use at both baseline and imaging assessments.

Liver MRI scans were acquired using a Siemens MAGNETOM Aera 1.5 T scanner (Syngo MR D13) and the LiverMultiScan protocol from Perspectum Ltd. (UK).^20^ Liver fat (%) was quantified using proton density fat fraction (PDFF). Liver steatosis was defined as liver fat ≥5%.^21^ Liver iron corrected T1 (cT1, measured in milliseconds [ms]) was also derived from liver MRI, which is highly correlated to liver inflammation and fibrosis.^22^, ^23^ MRI T1 relaxation time reflects extracellular fluid, which is characteristic of fibrosis and inflammation. The presence of iron, which can be determined from T2* maps, has an opposing effect. Combining T2* and T1 values can correct for this opposing effect, from which cT1 is derived. Higher cT1 values are associated with histological liver inflammation and fibrosis, although their relative contributions to the score are unclear.^24^ Liver fat content and cT1 have been recommended outcomes for assessing treatment‐induced histological improvements in people with metabolic dysfunction associated steatohepatitis.^25^

### 2.2 Covariates

Socio‐economic status was measured using the Townsend Deprivation Index, a postcode‐based social deprivation score. Self‐reported educational attainment was categorised as below secondary, lower secondary, upper secondary, vocational training and higher education. Self‐recorded ethnic background was classified into White or other.

Smoking status was self‐reported as current, previous or never smokers. Alcohol consumption was assessed via self‐reported intake of various alcoholic drinks; the consumption was summed up to derive average daily alcohol consumption (g/d). Physical activity level was measured with the International Physical Activity Questionnaire, and individuals were categorised into low, moderate and high levels, based on the frequency, duration and intensity of their physical activities.

Systolic and diastolic blood pressures were measured twice by trained staff, and the average was used for analysis. Blood biochemistry markers were measured at a central laboratory.^21^ Diabetes was defined as glycated haemoglobin (HbA1c) ≥39 mmol/mol and/or diagnosis of type 2 diabetes and/or on treatment for type 2 diabetes. Hypertension was defined as systolic blood pressure (BP) ≥130 and/or diastolic BP ≥85 mmHg and/or on antihypertensive drug treatment or diagnosis of hypertension. Body mass index (BMI) was calculated as weight (kg) divided by height squared (m^2^). High triglycerides (TG) were defined as plasma TG ≥1.70 mmol/L and/or on lipid lowering treatment. Low high‐density lipoprotein (HDL) cholesterol was defined as HDL‐cholesterol ≤1.0 mmol/L (male) (≤1.3 mmol/L [female]) and/or on lipid lowering treatment. Participants also reported whether they had any existing CVD, including angina, stroke and myocardial infarction. Use of statin and metformin were self‐reported. Since liver fat was not measured at baseline, we calculated fatty liver index (FLI) as a surrogate for liver fat at baseline assessment; FLI was calculated based on BMI, waist circumference, TG level and gamma‐glutamyl transpeptidase level.^26^

### 2.3 Statistical analysis

Participants' characteristics at imaging visit were summarised stratified by aspirin use status at imaging visit and by longitudinal pattern of aspirin use.

We fitted linear regression to examine the associations between aspirin use and longitudinal aspirin use pattern with liver fat and cT1, with beta and 95% confidence interval (CI) as effect measure. Logistic regression was fitted for liver steatosis, with odds ratio (OR) and 95% CI as effect measure. The models were adjusted for age, sex, ethnicity, Townsend Deprivation Index (in fifths), education, smoking, physical activity, daily alcohol consumption, BMI, diabetes, hypertension, high TG, low HDL, existing CVD and baseline FLI. The associations of aspirin use at baseline visit were adjusted for covariates measured at baseline, and the associations of aspirin use at imaging visit were adjusted for covariates measured at imaging visit.

We conducted subgroup analyses stratified by sex, age group (<65, ≥65), physical activity, alcohol drinking level (<20/30, 20/30–50/60, ≥50/60 g/d for female/male), BMI groups (<25, 25–30, ≥30 kg/m^2^), existing CVD, diabetes, hypertension, high TG, low HDL, and baseline FLI (<60, ≥60).

To further address confounding bias, we performed inverse probability of treatment weight (IPTW) analysis. First, we estimated propensity score using logistic regression models for aspirin use, adjusted for the covariates mentioned above. Second, we calculated IPTW based on the marginal probability of the observed exposure divided by the predicted propensity score. Third, we examined the overlap of propensity score distributions between groups to ensure adequate overlap. Fourth, we ran a weighted regression model of the outcome on aspirin use, using the calculated IPTW as weights. We fitted multinomial regression to generate propensity score for longitudinal aspirin use patterns. For sensitivity analysis, IPTW were truncated at the 0.1% and 99.9% percentiles to limit the influence of extreme values.

We also performed sensitivity analysis by including uses of statin and metformin in covariate adjustment in Cox models and in generating propensity score in IPTW analyses.

## 3 RESULTS

**FIGURE 1:**
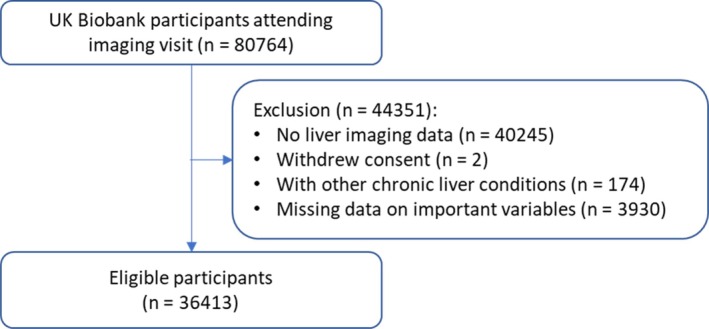
Flowchart of participant selection.

This study included 36 413 participants (mean age 64.6 years, 48.6% males; Figure 1). At imaging assessment, 3530 (9.7%) participants reported regular aspirin use. Compared to non‐users, aspirin users were more likely to be males, older, socio‐economically deprived, less educated, former or current smokers, and physically inactive, and to consume more alcohol. They also had higher baseline FLI, BMI, and systolic blood pressure, and higher prevalence of hypertension, diabetes, dyslipidaemia, as well as existing CVD. Aspirin users also had a higher prevalence of using statin and metformin. (Table 1).

**TABLE 1:** Participants' characteristics at imaging visit, stratified by aspirin use at imaging visit.

	Overall	Non‐user	Aspirin user	*p*‐Value
	*n* = 36 413 (100.0%)	*n* = 32 883 (90.3%)	*n* = 3530 (9.7%)	
Sex, male	17 683 (48.6%)	15 277 (46.5%)	2406 (68.2%)	<0.01
Age, years	64.6 (7.7)	64.2 (7.6)	67.4 (7.2)	<0.01
Townsend deprivation index				0.14
1st fifth (least deprived)	7283 (20%)	6618 (20.1%)	665 (18.8%)	
5th fifth (most deprived)	7239 (19.9%)	6497 (19.8%)	742 (21%)	
Education, higher education	17 728 (48.7%)	16 159 (49.1%)	1569 (44.4%)	<0.01
Ethnicity, White	35 267 (96.9%)	31 869 (96.9%)	3398 (96.3%)	0.04
Smoking, never	22 788 (62.6%)	20 902 (63.6%)	1886 (53.4%)	<0.01
Alcohol drinking, g/d^a^	10.3 (2.7, 20.6)	10.3 (2.7, 20.1)	11.4 (2.8, 22.8)	<0.01
Physical activity, high	14 519 (39.9%)	13 193 (40.1%)	1326 (37.6%)	0.02
Body mass index, kg/m^2^	26.5 (4.3)	26.4 (4.3)	27.4 (4.3)	<0.01
Waist circumference, cm	88.2 (12.5)	87.8 (12.5)	92.5 (12.1)	<0.01
Systolic blood pressure, mmHg	138.7 (18.5)	138.5 (18.5)	141.0 (18.5)	<0.01
Diastolic blood pressure, mmHg	78.7 (10.0)	78.7 (10.0)	78.1 (10.1)	<0.01
Fatty liver index baseline	41.1 (29.3)	40.0 (29.1)	51.1 (29.1)	<0.01
Hypertension	29 439 (80.8%)	26 256 (79.8%)	3183 (90.2%)	<0.01
Diabetes	5045 (13.9%)	4181 (12.7%)	864 (24.5%)	<0.01
High triglycerides	14 319 (39.3%)	12 529 (38.1%)	1790 (50.7%)	<0.01
Low HDL‐cholesterol	11 707 (32.2%)	10 256 (31.2%)	1451 (41.1%)	<0.01
Existing cardiovascular disease	422 (1.2%)	147 (0.4%)	275 (7.8%)	<0.01
Statin, %	3032 (8.3%)	2482 (7.5%)	550 (15.6%)	<0.01
Metformin, %	989 (2.7%)	749 (2.3%)	240 (6.8%)	<0.01
Liver steatosis	10 130 (27.8%)	8970 (27.3%)	1160 (32.9%)	<0.01
Liver fat, %	4.9 (4.9)	4.9 (4.9)	5.4 (5.2)	<0.01
Liver fat, %^a^	3.1 (2.2, 5.5)	3.0 (2.2, 5.4)	3.4 (2.4, 6.5)	<0.01
Liver cT1, ms	700.6 (54.9)	699.0 (54.5)	715.7 (56.0)	<0.01

*Note*: Diabetes was defined as glycated haemoglobin (HbA1c) ≥39 mmol/mol and/or diagnosis of type 2 diabetes and/or on treatment for type 2 diabetes. Hypertension was defined as systolic blood pressure (BP) ≥130 and/or diastolic BP ≥85 mmHg and/or on antihypertensive drug treatment or diagnosis of hypertension. High TG was defined as plasma TG ≥1.70 mmol/L and/or on lipid lowering treatment. Low HDL cholesterol was defined as HDL‐cholesterol ≤1.0 mmol/L (male) (≤1.3 mmol/L (female)) and/or on lipid lowering treatment.

Abbreviations: HDL: high density lipoprotein; TG, triglycerides.

^a^ Showing median (interquartile interval).

By longitudinal aspirin use patterns, 30 466 (83.7%) participants as never users, 1772 (4.9%) initiators, 2417 (6.6%) discontinuers, and 1758 (4.8%) persistent users. Across these groups, there was a graded increase in the likelihood of being male, older age, socio‐economically deprived, less educated, smokers, and having higher alcohol drinking, BMI, dyslipidaemia, and existing CVD. Discontinuers had slightly higher prevalence of diabetes than initiators. (Table S1, Supporting Information).

In multivariable‐adjusted analyses, aspirin use at baseline visit showed null associations with liver fat or cT1. However, aspirin use at imaging visit was inversely associated with liver fat (beta [95% CI]: −0.35 [−0.51, −0.20]), and odds of liver steatosis (OR [95% CI]: 0.83 [0.75, 0.90]). IPTW analyses yielded similarly negative associations with liver fat (−0.25 [−0.44, −0.05]) and liver steatosis (0.88 [0.80, 0.96]). By contrast, aspirin use was associated positively with higher liver cT1, with beta of 5.13 (3.23, 7.03) and 5.06 (2.41, 7.71) for multivariable adjustment analysis and IPTW analysis. (Table 2).

**TABLE 2:** Association between aspirin use at baseline and imaging visits and liver fat and cT1 (at imaging visit).

	Aspirin use at baseline visit	Aspirin use at imaging visit	
	Multivariable adjustment	Multivariable adjustment	IPTW
Liver fat, %	−0.05 (−0.22, 0.12)	−0.35 (−0.51, −0.20)	−0.25 (−0.44, −0.05)
Liver steatosis^a^	0.99 (0.90, 1.08)	0.83 (0.75, 0.90)	0.88 (0.80, 0.96)
Liver cT1, ms	6.29 (4.20, 8.39)	5.13 (3.23, 7.03)	5.06 (2.41, 7.71)

*Note*: Multivariable adjustment model included age, sex, deprivation index, ethnicity, physical activity, smoking, daily alcohol consumption, diabetes, hypertension, body mass index, high triglycerides, low high‐density lipoprotein, existing cardiovascular disease, and baseline fatty liver index. For association of aspirin use at baseline visit, the covariates were measured at baseline visit. for association of aspirin use at imaging visit, the covariates were measured at imaging visit.

Abbreviation: CI, confidence interval; IPTW, inverse probability of treatment weight.

^a^ Showing odds ratio (95 CI) for liver steatosis, and beta (95% CI) for liver fat and cT1.

**FIGURE 2:**
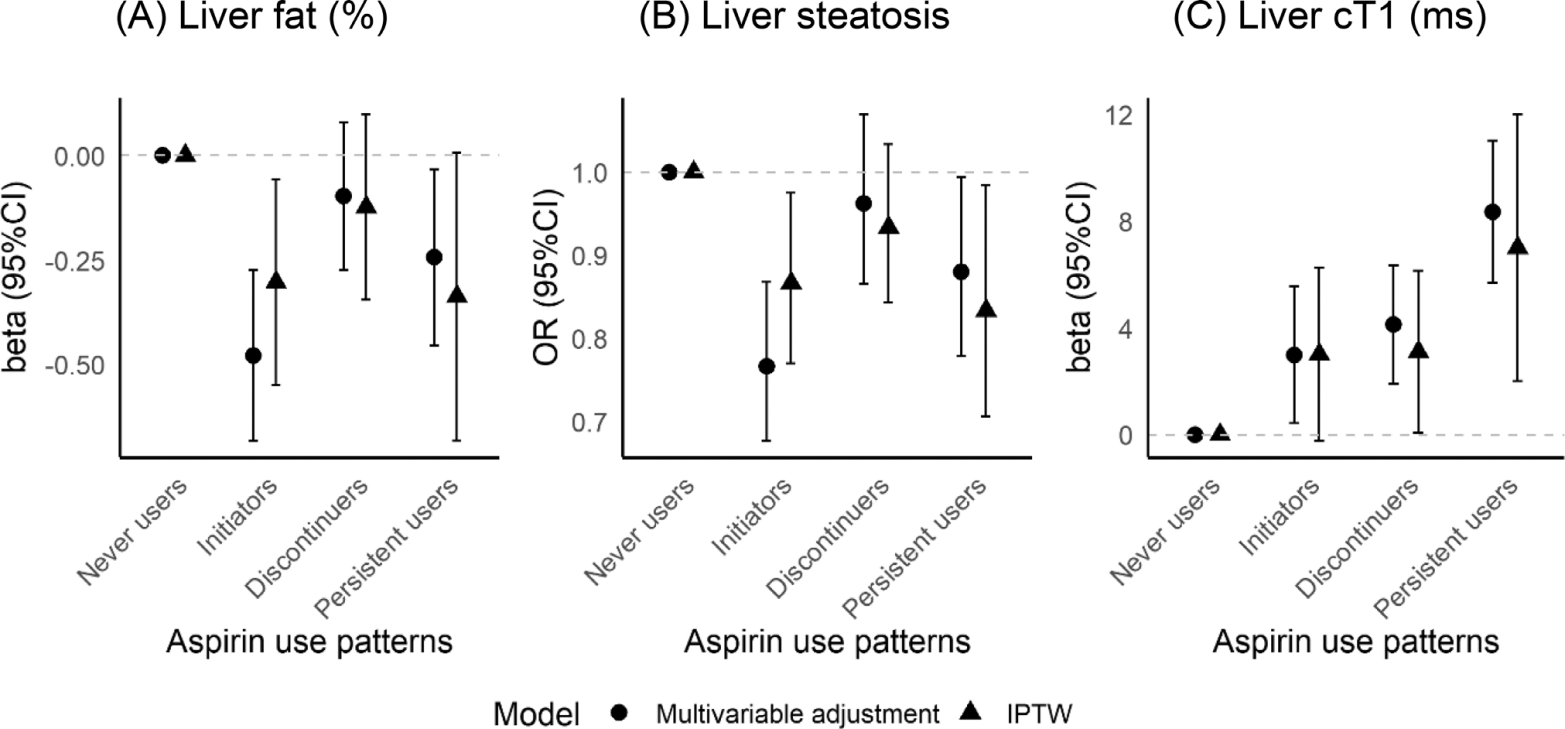
Associations between aspirin use patterns and liver fat, liver steatosis and cT1. Multivariable adjustment model included age, sex, deprivation index, ethnicity, physical activity, smoking, daily alcohol consumption, diabetes, hypertension, body mass index, high triglycerides, low high‐density lipoprotein, existing cardiovascular disease and baseline fatty liver index.

Comparing with never users in multivariable adjustment analysis, initiators and persistent users demonstrated lower liver fat (−0.48 [−0.68, −0.27] and −0.24 [−0.45, −0.03], respectively), while discontinuers showed a non‐significant association (−0.10 [−0.27, 0.08]). Similarly, initiators and persistent users showed a lower prevalence of liver steatosis (0.77 [0.68, 0.87], 0.88 [0.78, 0.99]), while discontinuers showed a non‐significant association (0.96 [0.87, 1.07]). Initiators, discontinuers and persistent users progressively showed higher cT1 (2.99 [0.44, 5.55], 4.12 [1.91, 6.33], 8.35 [5.70, 11.01]). IPTW analyses showed similar results. Compared to never users, initiators had lower liver fat (−0.30 [−0.55, −0.06]), persistent users had marginally non‐significant associations (−0.34 [−0.68, 0.01]), while discontinuers showed null associations (−0.12 [−0.35, 0.10]). Initiators and persistent users showed lower odds of liver steatosis (0.87 [0.77, 0.98] and 0.83 [0.71, 0.98]), while discontinuers showed non‐significant associations (0.93 [0.84, 1.03]). Compared with never users, initiators, discontinuers and persistent users progressively showed higher cT1 (3.01 [−0.23, 6.25], 3.11 [0.08, 6.14], 7.01 [2.02, 12.00]), in IPTW analysis. (Figure 2).

Subgroup analyses showed consistently negative associations between aspirin use at imaging visit and liver fat, and positive associations with liver cT1. Notably, the inverse association between aspirin use and liver fat was stronger among participants aged >65 years compared to those <65 years (−0.44 [−0.63, −0.26] vs. −0.08 [−0.35, 0.19], *p* for interaction <0.01) (Table S2). Sensitivity analysis using truncated IPTW produced similar results to primary IPTW analysis (Table S3).

The sensitivity analyses with additional adjustment for the uses of statin and metformin in Cox models and IPTW analyses generated similar results to the primary findings (Table S4). For example, use of aspirin was associated with reduced liver fat by 0.32 (0.47, 0.17) and increased liver cT1 by 5.11 (3.21, 7.02).

## 4 DISCUSSION

In this cohort of 36 413 participants, we found that both cross‐sectional and longitudinal aspirin use were associated with lower liver fat and higher liver cT1. Multivariable adjustment and IPTW analyses showed consistent results, and the associations were robust across subgroups.

The observed inverse associations between aspirin use and liver fat percentage align with previous evidence. Lonardo and Zheng^13^ reviewed epidemiological evidence supporting the antisteatotic effects of aspirin. Recent trial evidence has also shown low‐dose aspirin reduced liver fat content, although in a small sample.^16^ Shen et al.^10^ found that aspirin use was associated with lower NAFLD prevalence, especially in older adults, consistent with our findings of stronger associations in people >65 years old. Using UKB data, Vell et al.^27^ showed similar findings that aspirin was associated with lower incidence of liver disease in men, although they did not examine quantitative liver fat or disease activity. However, Huang et al.^14^ found a positive association between aspirin and SLD incidence, though their study was limited by outcome definition of using only clinical diagnosis captured in medical records, introducing misclassification bias.^17^ It is worth noting that the UKB imaging cohort had a mean liver fat of 4.9%, close to the diagnostic threshold for steatosis (≥5%), with only 27.8% of the participants exceeding this threshold. Although the observed aspirin‐associated liver fat reduction was modest in absolute terms (0.35%), a shift of this magnitude could meaningfully alter the proportion of individuals classified as having steatosis at the population level, for example, to 30.1% in this cohort.

The magnitude of liver fat reduction associated with aspirin use in this study was modest. While previous clinical trial data suggest that a 30% relative reduction in liver fat is associated with a substantially higher likelihood of histological improvement, this threshold was derived from interventional studies in selected patient populations receiving liver‐directed therapies.^25^ In our population‐based cohort, baseline liver fat levels were relatively low, and aspirin use may vary regarding dose and duration between users. Therefore, a smaller relative reduction in liver fat (approximately 7%–10%) is expected and likely reflects modest metabolic effects rather than histological resolution. These findings should be interpreted in the context of population‐level risk modulation rather than individual‐level treatment response. Accordingly, the observed PDFF differences should not be interpreted as evidence of clinically meaningful histological improvement.

Several mechanisms may underlie the observed associations. Aspirin exerts both systematic anti‐inflammatory effects and organ‐specific effects, include promoting mitochondrial biogenesis via PGC‐1α, attenuating hepatic collagen production through TGF‐β1 inhibition, and suppressing platelet activation and pro‐inflammatory signalling.^13^ Preclinical studies show that platelets infiltrate the liver and promote inflammatory via Kupffer cells activation; whereas aspirin inhibits proinflammatory cyclooxygenase‐2 and platelet derived growth factor signalling.^16^ In animal models, aspirin reduced obesity and liver fat and improved glucose intolerance,^28^ and modulated the PPAR‐AMPK‐PGC‐1α pathway in dyslipidaemic states.^29^

By contrast, we found that aspirin use was associated with increased liver cT1 by 5 ms, a biomarker of hepatic inflammation and fibrosis activity. This finding seems contradictory to some prior studies. Simon et al.^11^ and Jiang et al.^12^ reported that daily aspirin use was associated with reduced fibrosis in individuals with MASLD, assessed using non‐invasive fibrosis scores, including fibrosis‐4, NAFLD fibrosis score and AST/platelet ratio, although another study^15^ found null associations. Importantly, however, the observed increase in cT1 in our study was minimal (5 ms), well below the clinically meaningful threshold of 80 ms.^25^ Therefore, while statistically significant, this difference has limited clinical relevance and should not outweigh the potential antisteatotic benefits of aspirin.

An exploratory analysis at UK Biobank baseline assessment revealed that aspirin use was associated with a slightly higher FIB‐4 score, with a beta of 0.03 (95% CI 0.01, 0.05) after adjustment for age, sex, socio‐economic status, lifestyle, cardiometabolic risk factors, and uses of statin and metformin. The magnitude of this difference was small and unlikely to be clinically meaningful, similar to our MRI‐based findings.

Notably, many prior studies reporting protective effects of aspirin against fibrosis relied on blood‐based surrogate scores (e.g., fibrosis 4, NAFLD fibrosis score, AST/platelet ratio), which largely reflect systemic biochemical correlates of liver injury and are sensitive to changes in hepatic necroinflammation and transaminase fluctuations. By contrast, cT1 captures local fibroinflammatory changes, oedema and extracellular matrix, and may be less responsive to short‐term metabolic improvements. Therefore, a plausible interpretation is that aspirin reduce hepatocellular fat accumulation and mitigate metabolic injury, but is less likely to rapidly reverse established imaging‐detectable fibroinflammatory changes.

Alternative explanations remain possible. For example, aspirin users often have greater cardiometabolic burden, which may disproportionately influence cT1 and cause residual confounding. Temporal differences may also play a role. Antisteatotic effects of aspirin may occur earlier, while fibroinflammatory injury processes captured by cT1 progress over longer periods. Together, these findings suggest that aspirin reduces steatosis but does not necessarily rapidly protect against liver inflammation or fibrosis. Future investigations are needed to further examine the causal effects, with clear temporality, dose response relationship, and underlying mechanisms.

The strengths of this study include accurate quantitative measure of liver fat and cT1 via liver MRI, a large sample size, assessment of both cross‐sectional and longitudinal aspirin use, and the application of multivariable adjustment and IPTW methods, which strengthen causal inference. However, there are also some limitations. First, aspirin use was self‐reported as a binary variable, without information on dose and duration, precluding dose–response analyses. Second, intermittent aspirin use patterns occurring between UK Biobank baseline and imaging assessments could not be fully characterised due to limited availability of repeat assessment data, and such patterns were therefore not explicitly modelled. Third, baseline liver MRI measures were unavailable, preventing direct evaluation of longitudinal changes in liver fat or cT1 over time; although we adjusted for baseline FLI, residual confounding from unmeasured liver changes may remain. Future longitudinal studies with repeated measures of the outcomes before and after treatment are warranted to examine the causality of the observed associations. Fourth, although we adjusted for a wide range of covariates, residual confounding cannot be entirely excluded. Of note, participants who started aspirin were likely to comply with other medical advice, such as lifestyle modification, etc. Application of multivariable adjustment and IPTW methods strengthened the result validity. Fifth, UKB data have restricted representativeness to the general UK population, especially on ethnicity, health status and socio‐economic status, which may limit the generalizability of our findings to other populations. Finally, while MRI measures are highly informative, cT1 is an indirect measure of inflammation and fibrosis, and may not perfectly reflect liver fibroinflammatory injury. Biopsy remains the gold standard for quantifying steatosis and fibrosis status.

## 5 CONCLUSION

In summary, this large cohort study showed that aspirin use was associated with lower liver fat. The reduction in liver fat supports previous epidemiological and mechanistic evidence of aspirin's potential antisteatotic effects, while the minimal increase in cT1 is not clinically meaningful. Future mechanistic and longitudinal studies with detailed exposure data, dose–response analyses, and direct measures of fibrosis progression are warranted to clarify aspirin's role in the prevention and management of liver steatosis.

## AUTHOR CONTRIBUTIONS

Qi Feng conceived the research idea, performed primary analysis and drafted the manuscript. All authors conducted data analysis, interpreted results, critically reviewed and revised the manuscript.

## CONFLICT OF INTEREST STATEMENT

CI has conducted consultancy work for Novo Nordisk outside the submitted work. The other authors declare no conflict of interest. [Correction added on 3 March 2026 after first online publication: The conflict of interest has been amended in this version.]

## Supporting information


**Table S1:** Participants' characteristics at imaging visit stratified by longitudinal pattern of aspirin use.
**Table S2:** Subgroup analysis for associations between aspirin use at imaging visit and liver fat and liver cT1.
**Table S3:** Sensitivity analysis for associations between aspirin use, aspirin use patterns and liver fat, liver steatosis, and liver cT1 using truncated IPTW weights.
**Table S4:** Association between aspirin use at baseline and imaging visits and liver fat and cT1 (at imaging visit) with additional adjustment of use of statin and metformin.

## Data Availability

UK Biobank data are available at https://www.ukbiobank.ac.uk/.
