# Supplementary material for: Associations between use of aspirin and magnetic resonance imaging‐derived liver fat and fibroinflammation
Source: Diabetes Obes Metab. 2026 Feb 18;28(5):3895–902. doi: 10.1111/dom.70570 (PMC13071233; doi:10.1111/dom.70570)
Supplement: Supplementary file 1 — Table S1: Participants' characteristics at imaging visit stratified by longitudinal pattern of aspirin use. Table S2: Subgroup analysis for associations between aspirin use at imaging visit and liver fat and liver cT1. Table S3: Sensitivity analysis for associations between aspirin use, aspirin use patterns and liver fat, liver steatosis, and liver cT1 using truncated IPTW weights. Table S4: Association between aspirin use at baseline and imaging visits and liver fat and cT1 (at imaging visit) with additional adjustment of use of statin and metformin. [file DOM-28-3895-s001.docx]

Associations between use of aspirin and MRI-derived liver fat and fibroinflammation

Supplementary table 1: Participants’ characteristics at imaging visit stratified by longitudinal pattern of aspirin use

|  | Never user | Initiator | Discontinuer | Persistent user |
| --- | --- | --- | --- | --- |
|  | n = 30466  (83.7%) | n = 1772  (4.9%) | n = 2417  (6.6%) | N = 1758  (4.8%) |
| Sex, male | 13825 (45.4%) | 1131 (63.8%) | 1452 (60.1%) | 1275 (72.5%) |
| Age, years | 64.0 (7.6) | 66.1 (7.4) | 67.6 (7.5) | 68.7 (6.8) |
| Townsend Deprivation Index |  |  |  |  |
| 1st fifth (least deprived) | 6148 (20.2%) | 350 (19.8%) | 470 (19.4%) | 315 (17.9%) |
| 5th fifth (most deprived) | 6015 (19.7%) | 354 (20.0%) | 482 (19.9%) | 388 (22.1%) |
| Education, higher education | 15009 (49.3%) | 805 (45.4%) | 1150 (47.6%) | 764 (43.5%) |
| Ethnicity, White | 29526 (96.9%) | 1706 (96.3%) | 2343 (96.9%) | 1692 (96.2%) |
| Smoking, never | 19552 (64.2%) | 1000 (56.4%) | 1350 (55.9%) | 886 (50.4%) |
| Alcohol drinking, g/d* | 10.2 (2.7, 20.0) | 11.0 (2.4, 22.2) | 10.3 (2.4, 21.0) | 11.9 (3.2, 23.5) |
| Physical activity, high | 12327 (40.5%) | 685 (38.7%) | 866 (35.8%) | 641 (36.5%) |
| Body mass index, kg/m^2^ | 26.3 (4.3) | 27.1 (4.3) | 27.3 (4.4) | 27.7 (4.2) |
| Waist circumference, cm | 87.5 (12.4) | 91.3 (12.0) | 92.1 (12.8) | 93.9 (12.1) |
| Systolic blood pressure, mmHg | 138.2 (18.5) | 139.7 (18.2) | 141.7 (19.2) | 142.5 (18.7) |
| Diastolic blood pressure, mmHg | 78.7 (10.0) | 78.1 (10.1) | 78.8 (10.3) | 78.1 (10.1) |
| Fatty liver index baseline | 39.3 (28.9) | 48.3 (29.2) | 49.4 (29.6) | 53.8 (28.9) |
| Hypertension | 24133 (79.2%) | 1551 (87.5%) | 2123 (87.8%) | 1632 (92.8%) |
| Prediabetes/diabetes | 3608 (11.8%) | 322 (18.2%) | 573 (23.7%) | 542 (30.8%) |
| High triglycerides | 11367 (37.3%) | 912 (51.5%) | 1162 (48.1%) | 878 (49.9%) |
| Low HDL-cholesterol | 9359 (30.7%) | 700 (39.5%) | 897 (37.1%) | 751 (42.7%) |
| Existing cardiovascular disease | 97 (0.3%) | 100 (5.6%) | 50 (2.1%) | 175 (10.0%) |
| Statin, % | 2159 (7.1%) | 282 (15.9%) | 323 (13.4%) | 268 (15.2%) |
| Metformin, % | 559 (1.8%) | 75 (4.2%) | 190 (7.9%) | 165 (9.4%) |
| Liver fat, % | 4.8 (4.9) | 5.1 (5.1) | 5.6 (5.3) | 5.8 (5.3) |
| Liver fat, %* | 3.0 (2.1, 5.3) | 3.1 (2.3, 5.8) | 3.6 (2.4, 6.7) | 3.7 (2.5, 7.1) |
| Liver cT1, | 698.1 (54.2) | 710.2 (56.1) | 713.0 (58.2) | 722.1 (55.5) |
| Liver steatosis | 8142 (26.7%) | 526 (29.7%) | 828 (34.3%) | 634 (36.1%) |

HDL: high density lipoprotein. Diabetes was defined as glycated haemoglobin (HbA1c) ≥39 mmol/mol and/or diagnosis of type 2 diabetes and/or on treatment for type 2 diabetes. Hypertension was defined as systolic blood pressure (BP) ≥130 and/or diastolic BP ≥ 85 mmHg and/or on antihypertensive drug treatment or diagnosis of hypertension. High TG was defined as plasma TG ≥1.70 mmol/L and/or on lipid lowering treatment. Low HDL cholesterol was defined as HDL-cholesterol ≤1.0 mmol/L (male) (≤1.3 mmol/L (female)) and/or on lipid lowering treatment. *: showing median (interquartile interval).

Supplementary table 2: subgroup analysis for associations between aspirin use at imaging visit and liver fat and liver cT1

| Subgroup | n | Liver fat | P for subgroup difference | Liver cT1 | P for subgroup difference |
| --- | --- | --- | --- | --- | --- |
| **Sex** |  |  |  |  |  |
| Female | 18730 | -0.39 (-0.64, -0.15) |  | 3.37 (0.14, 6.59) |  |
| Male | 17683 | -0.34 (-0.54, -0.13) | 0.67 | 5.53 (3.10, 7.95) | 0.15 |
| **Age, years** |  |  |  |  |  |
| < 65 | 17335 | -0.08 (-0.35, 0.19) |  | 5.07 (1.67, 8.46) |  |
| ≥ 65 | 19078 | -0.44 (-0.63, -0.26) | < 0.01 | 5.16 (2.85, 7.46) | 0.88 |
| **Physical activity** |  |  |  |  |  |
| Low | 3537 | -0.14 (-0.74, 0.45) |  | 7.28 (0.52, 14.04) |  |
| Moderate | 13001 | -0.38 (-0.64, -0.12) | 0.42 | 4.78 (1.58, 7.98) | 0.41 |
| High | 14519 | -0.32 (-0.55, -0.10) | 0.27 | 5.79 (2.82, 8.76) | 0.47 |
| **Alcohol drinking** |  |  |  |  |  |
| Low | 30079 | -0.30 (-0.46, -0.13) |  | 5.32 (3.21, 7.43) |  |
| Intermediate | 5436 | -0.53 (-0.96, -0.10) | 0.61 | 4.97 (0.08, 9.87) | 0.64 |
| High | 898 | -0.77 (-2.07, 0.54) | 0.41 | 1.26 (-12.14, 14.65) | 0.34 |
| **Existing CVD** |  |  |  |  |  |
| No | 35991 | -0.34 (-0.49, -0.18) |  | 5.27 (3.34, 7.20) |  |
| Yes | 422 | -0.54 (-1.46, 0.38) | 0.34 | -3.28 (-22.94, 16.38) | 0.37 |
| **Diabetes** |  |  |  |  |  |
| No | 31368 | -0.33 (-0.49, -0.16) |  | 5.41 (3.28, 7.55) |  |
| Yes | 5045 | -0.40 (-0.84, 0.03) | 0.86 | 4.46 (-0.11, 9.02) | 0.72 |
| **Hypertension** |  |  |  |  |  |
| No | 6974 | -0.06 (-0.37, 0.25) |  | 3.02 (-2.34, 8.37) |  |
| Yes | 29439 | -0.39 (-0.57, -0.22) | 0.28 | 5.23 (3.16, 7.30) | 0.16 |
| **Body mass index, kg/m^2^** |  |  |  |  |  |
| < 25 | 14736 | -0.13 (-0.27, -0.01) |  | 5.45 (2.52, 8.37) |  |
| 25-30 | 15129 | -0.38 (-0.62, -0.15) | 0.64 | 6.30 (3.52, 9.09) | 0.24 |
| ≥30 | 6483 | -0.63 (-1.15, -0.10) | 0.20 | 1.33 (-3.66, 6.32) | 0.13 |
| **High triglycerides** |  |  |  |  |  |
| No | 22094 | -0.21 (-0.39, -0.03) |  | 6.40 (3.82, 8.98) |  |
| Yes | 14319 | -0.45 (-0.72, -0.18) | 0.06 | 3.96 (1.04, 6.87) | 0.14 |
| **Low HDL** |  |  |  |  |  |
| No | 24706 | -0.24 (-0.42, -0.06) |  | 6.10 (3.71, 8.49) |  |
| Yes | 11707 | -0.48 (-0.76, -0.19) | 0.36 | 3.73 (0.47, 6.98) | 0.42 |
| **Fatty liver index (baseline)** |  |  |  |  |  |
| < 60 | 25575 | -0.31 (-0.46, -0.15) |  | 6.53 (4.21, 8.84) |  |
| ≥60 | 10785 | -0.42 (-0.75, -0.08) | 0.43 | 3.04 (-0.40, 6.49) | 0.04 |

Multivariable adjustment model included age, sex, deprivation index, ethnicity, physical activity, smoking, daily alcohol consumption, diabetes, hypertension, body mass index, high triglycerides, low high-density lipoprotein, existing cardiovascular disease, and baseline fatty liver index.

Supplementary table 3: Sensitivity analysis for associations between aspirin use, aspirin use patterns and liver fat, liver steatosis, and liver cT1 using truncated IPTW weights

|  |  | Longitudinal aspirin use pattern | | | |
| --- | --- | --- | --- | --- | --- |
|  | Aspirin use at imaging visit | Never user | initiator | discontinuer | Persistent user |
| Liver fat, % | -0.22 (-0.42, -0.03) | Reference | -0.30 (-0.54, -0.05) | -0.12 (-0.34, 0.10) | -0.19 (-0.51, 0.13) |
| Liver steatosis* | 0.89 (0.81, 0.97) | Reference | 0.87 (0.77, 0.98) | 0.94 (0.84, 1.04) | 0.89 (0.76, 1.04) |
| Liver cT1, ms | 5.21 (2.59, 7.83) | Reference | 3.08 (-0.16, 6.31) | 3.17 (0.15, 6.20) | 8.71 (4.32, 13.11) |

Multivariable adjustment model included age, sex, deprivation index, ethnicity, physical activity, smoking, daily alcohol consumption, diabetes, hypertension, body mass index, high triglycerides, low high-density lipoprotein, existing cardiovascular disease, and baseline fatty liver index. *: showing odds ratio (95CI) for liver steatosis, and beta (95%CI) for liver fat and cT1.

Supplementary table 4: Association between aspirin use at baseline and imaging visits and liver fat and cT1(at imaging visit) with additional adjustment of use of statin and metformin

|  | Aspirin use at baseline visit |  | Aspirin use at imaging visit | |
| --- | --- | --- | --- | --- |
|  | Multivariable  adjustment |  | Multivariable adjustment | IPTW |
| Liver fat, % | -0.04 (-0.21, 0.13) |  | -0.32 (-0.47, -0.17) | -0.21 (-0.39, -0.01) |
| Liver steatosis* | 0.99 (0.90, 1.09) |  | 0.84 (0.77, 0.92) | 0.88 (0.80, 0.98) |
| Liver cT1, ms | 6.06 (3.94, 8.18) |  | 5.11 (3.21, 7.02) | 4.64 (1.77, 7.51) |

Multivariable adjustment model included age, sex, deprivation index, ethnicity, physical activity, smoking, daily alcohol consumption, diabetes, hypertension, body mass index, high triglycerides, low high-density lipoprotein, existing cardiovascular disease, use of statin and metformin, and baseline fatty liver index. For association of aspirin use at baseline visit, the covariates were measured at baseline visit. for association of aspirin use at imaging visit, the covariates were measured at imaging visit. *: showing odds ratio (95CI) for liver steatosis, and beta (95%CI) for liver fat and cT1.
